# Prevalence of Obstructive Lung Disease in Asymptomatic Gas Field Workers

**DOI:** 10.7759/cureus.3580

**Published:** 2018-11-12

**Authors:** Fatima Zaina, Salva Arshad, Jawed Abubaker, Arsalan Ahmed, Musa Karim

**Affiliations:** 1 Pulmonology, Dr. Ziauddin University and Hospital, Karachi, PAK; 2 Pulmonology, National Institute of Cardiovascular Diseases (NICVD), Karachi, PAK; 3 Internal Medicine, Dr. Ziauddin University and Hospital, Karachi, PAK; 4 Miscellaneous, National Institute of Cardiovascular Diseases (NICVD), Karachi, PAK

**Keywords:** spirometry, obstructive lung disease, oil and gas

## Abstract

Introduction

Obstructive lung disease, if not managed appropriately and in a timely manner, increases morbidity and mortality. The aim of this study was to see an obstruction on spirometry reports of clinically asymptomatic oil and gas field workers.

Methods

In this retrospective observational study, spirometry reports performed at the pulmonary function laboratory of Ziauddin Hospital and University, Karachi, were reviewed. All reports were of the clinically asymptomatic employees of an oil and gas company in Pakistan, who presented for their routine assessment. Obstructive impairment was defined as a forced expiratory volume in one second (FEV1) to forced vital capacity (FVC) ratio of less than 0.7.

Results

Of the total of 199 spirometry reports, 197 (99%) were of male employees. The mean age of the employees was 30.52 ± 8.24 years and 46 (23.1%) employee were smokers. Obstruction was observed in 48 (24.1%) of the reports of the employees with 13 (27.1%) smokers and 35 (72.9%) non-smokers. No statistically significant association between obstruction and gender, age, and smoking was observed.

Conclusion

In this study, we observed obstructive impairment in around one-fourth of the otherwise clinically asymptomatic oil and gas field workers with no apparent impact of baseline smoking behavior.

## Introduction

Chronic obstructive pulmonary disease (COPD), largely irreversible, is a progressive airflow obstruction predominantly attributed to smoking in the middle to late age population segment [1]. Smoking is considered to be the only risk factor of COPD, however, mounting evidence suggestive of other etiologically important factors, which, either alone or in combination with smoking, relate to the COPD [1-2]. Among other etiological and environmental factors that are attributable to COPD, occupational exposure is reported to be responsible for at least 15% to 20% of all cases of COPD [3-5].

Occupational exposure to gas, fumes, and organic and inorganic dust is an underestimated group of obstructive lung diseases (asthma and chronic obstructive pulmonary disease) [6-7]. Multiple, randomized controlled trials showed that exposure to workplace dust and fumes is associated with increased airflow limitation, emphysema, and gas trapping, as demonstrated by a computed tomography (CT) scan, in both men and women [8]. National Health and Nutrition Examination Survey III, a large U.S. population-based survey of almost 10,000 adults aged 30-75 years, anticipated that the portion of COPD attributable to workplace exposures was 19.2% in general and 31.1% among never-smokers [9].

Obstructive lung diseases are underestimated because they usually present clinically when they are slightly advanced. An early diagnosis of the disease is crucial in treating, preventing, and decreasing the disease burden, which is increasing in a threatening manner. Globally, aggressive measures are being taken to prevent the disease so that the disease burden itself goes down. In this regard, many diagnostic tools have been used for the detection of the disease, among which the pulmonary function test (PFT) has a pivotal role [10]. Early detection of obstructive impairment can be done confidently with PFTs [11-12]. The aim of this study was to see any abnormality in a selected population that works in oil- and gas-exposed areas and are clinically asymptomatic.

## Materials and methods

After taken the permission of the ethical review committee of the institution for this retrospective observational study, a total of 199 spirometry reports were reviewed, which were performed at the pulmonary function laboratory at Ziauddin Hospital and University, Karachi, during the period August 2015 to July 2016. All reports were of the clinically asymptomatic employees of an oil and gas company in Pakistan, who presented for their routine assessment. Spirometry reports of employees with a prior history of lung disease were excluded. Spirometry was performed by trained and certified staff using the portable Vitalograph (Kansas, USA) compact expert model number: 6600. Maneuvers were performed thrice for forced vital capacity (FVC), forced expiratory volume in one second (FEV1), and ratio FEV1/FVC for the diagnosis of obstruction. Obstructive impairment was defined as an FEV1 to FVC ratio of less than 0.7. The participants whose results came out to be abnormal were given a short-acting beta agonist (Salbutamol 200 mcg) and repeat spirometry was done for the post-bronchodilator change. A predefined structural questionnaire was used for the retrieval of required information, including age, gender, smoking status, family history of asthma, and obstruction as per spirometry. IBM SPSS Statistics for Windows, Version 21.0. (IBM Corp., Armonk, NY, US) was used to enter and analyze the collected data. Mean ± standard deviation (SD) for continuous variables, like age in years, frequency, and percentages, were calculated for categorical variables. The Chi-square test was applied to evaluate the association between categorical variables. A two-sided p-value ≤ 0.05 was taken as the criteria for statistical significance.

## Results

Of the total of 199 spirometry reports, 197 (99%) were of male employees and only 2 (1%) were of female employees. The mean age of the employees was 30.52 ± 8.24 years, with 116 (58.3%) employees between 18 and 30 years of age. None of the employees had a family history of asthma and 46 (23.1%) employees were smokers. The baseline characteristics of the employees are summarized in Table 1.

**Table 1:** Baseline characteristics

Characteristics	Frequency	Percentage
Male	197	99.0%
Female	2	1.0%
Age (mean ± SD)	30.52 ± 8.24 years	
18 to 30 years	116	58.3%
More than 30 years	83	41.7%
Smoker	46	23.1%
Family History - Asthma	0	0.0%

Of all the asymptomatic employees, 48 (24.1%) were found to have an obstruction, out of which 13 (27.1%) were smokers and 35 (72.9%) were non-smokers. No statistically significant association between obstruction and gender, age, and smoking was observed with chi-square p-values of 0.432, 0.094, and 0.454, respectively. The presence of an obstruction by the baseline characteristics of the employees is presented in Table 2.

**Table 2:** Obstruction by employees baseline characteristics **p-values are based on the chi-square test

Characteristics Frequency (row %)	Obstruction		**p-value
	Yes	No	
Overall	48 (24.1%)	151 (75.9%)	-
Gender			
Male	48 (24.4%)	149 (75.6%)	0.423
Female	0 (0.0%)	2 (100.0%)	
Age			
18 to 30 years	23 (19.8%)	93 (80.2%)	0.094
More than 30 years	25 (30.1%)	58 (69.9%)	
Smoking			
Smokers	13 (28.3%)	33 (71.7%)	0.454
Non-smokers	35 (22.9%)	118 (77.1%)	

## Discussion

The aim of this study was to evaluate abnormal spirometry in individuals who were otherwise healthy and presented for screening as a part of their required assessment. As the data we selected was of a population that worked in the oil and gas fields, we anticipated that these recruits will have some level of airway obstruction/reactive airway disease. In order to do this, we observed 199 spirometry reports done as an outpatient in our pulmonary function laboratory.

Our data demonstrated that 48 (24.1%) employees demonstrated an obstruction/reactive airway disease as detected by spirometry, with no apparent impact of baseline characteristics, such as gender, age, and smoking status. Several studies across the globe have established that occupational exposure to vapors, gases, dust, or fumes (VGDF), to a certain extent, is the cause of COPD development [1,5,13-17]. The extent of COPD due to occupational exposure varies from 15% to 20% [3-5,17]. These variations are partly due to varying occupational categories and the diagnostic criteria of COPD. The results of our study for oil and gas field workers are slightly higher than those reported in past studies. There are uncertainties in the literature about certain occupational exposures: whether it increases the risk of COPD independently or in combination with smoking [1]. Owing to the fact that smoking is the only and most important risk factor of COPD, it is important to address the potential modification effect of smoking while studying any underlying occupational relationship with COPD. In our study, obstructions among smokers and nonsmokers were observed to be 28.3% vs. 22.9%, respectively, with a chi-square p-value of 0.454 (statistically insignificant). This insignificant association of smoking with obstruction, assessed by spirometry, does reveal the potential evidence that occupational exposure among the oil and gas workers could enhance the risk of COPD regardless of baseline smoking behavior. Similarly, no significant association of obstruction was observed with age but the percentage of obstruction was relatively higher among employees more than 30 years of age, 30.1% vs. 19.8% with the p-value of 0.094, as compared to employees of age between 18 and 30 years. This relatively higher rate can be attributed to the fact that employees of relatively older ages are supposed to have more years of exposure to gas, fumes and organic and inorganic dust.

After reviewing the data, screening at the time of recruiting individuals for a job that requires a risk of exposure to a noxious material is a dire need. We need protocols to periodically screen the oil and gas field workers for any reactive airway disease due to exposure to the hazardous material. This information is important in either providing protection or preventing the disease in workers in the exposed environment. As there are no protocols for the screening of persons who are working or about to work in an exposed area, this study will help in developing some guidelines for recruitment. In future also, this subject can be further evaluated by following the patients prospectively to look for the effects of the noxious stimuli they have been exposed to.

The retrospective design, small sample size, and single oil and gas company coverage may limit the generalizability of the research findings.

## Conclusions

In this study, we observed an obstructive impairment in around one-fourth of the otherwise clinically asymptomatic oil and gas field workers, with no apparent impact of baseline smoking behavior. We suggest screening protocols should be made for the recruitment of new employees and a periodic assessment of existing oil and gas field workers be put in place so that better protective and preventive strategies can be formed. Such initiatives will help us decrease the overall burden of disease in our society.
